# Time to treatment initiation and its impact on real‐world survival in metastatic colorectal cancer and pancreatic cancer

**DOI:** 10.1002/cam4.5133

**Published:** 2022-08-17

**Authors:** Olumide Gbolahan, Neda Hashemi‐Sadraei, Suri Yash, Grant Williams, Rekha Ramachandran, Young‐il Kim, Ravikumar Paluri, Darryl Outlaw, Bassel El‐Rayes, Lisle Nabell

**Affiliations:** ^1^ Birmingham School of Medicine and O'Neal Comprehensive Cancer Center University of Alabama Birmingham Alabama USA; ^2^ University of New Mexico School of Medicine New Mexico USA; ^3^ Division of Preventive Medicine University of Alabama School of Medicine Birmingham Alabama USA; ^4^ Wake Forest School of Medicine North Carolina United States; ^5^ Emory University School of Medicine, and Winship Cancer Institute Georgia

**Keywords:** colorectal neoplasms, decision making, outcome assessment, pancreatic neoplasms, time‐to‐treatment

## Abstract

**Background:**

Given the dearth of data regarding the time to treatment initiation (TTI) in the palliative setting, and its impact on survival outcomes, we sought to determine TTI in a real‐world cohort of metastatic colorectal cancer (mCRC) and metastatic pancreatic cancer (mPC) patients and evaluate the impact of TTI on real‐world survival outcomes.

**Methods:**

We collected survival and treatment data for mCRC and mPC from the Flatiron Health electronic health records (EHR) derived database. We divided TTI into 3 categories: < 2 weeks, 2–< 4 weeks, and 4–8 weeks, from diagnosis to first‐line therapy. Outcome measures were median TTI, real‐world overall survival (RW‐OS) based on TTI categories by Kaplan–Meier method, and impact of TTI on survival using cox proportional hazard models.

**Results:**

Among 7108 and 3231 patients with mCRC and mPC treated within 8 weeks of diagnosis, the median TTI were 28 days and 20 days. Median RW‐OS for mCRC was 24 months; 26.9 months versus 22.6 and 18.05 months in the 4–8‐week, 2–< 4 week (control) and < 2‐week groups, respectively (*p* < 0.0001). For mPC, median RW‐OS was 8 months, without significant difference in RW‐OS among the groups (*p* = 0.05). The 4–8‐week group was associated with lower hazard of death (HR 0.782, 95% CI 0.73–0.84, *p* < 0.0001) and the < 2‐week group was associated with a higher hazard of death (HR 1.26, 95% CI 1.15–1.38, *p* < 0.0001) in mCRC. The 4–8‐week group was associated with lower hazard of death for mPC (HR 0.88, 95% CI 0.8–0.97, *p* = 0.0094).

**Conclusion:**

In a real‐world cohort of patients treated within 8 weeks of diagnosis, and with the limitations of a retrospective study, later TTI did not have a negative impact on survival outcomes in mCRC and mPC.

## Graphical abstract

In a real‐world cohort of patients who received treatment within 8 weeks of diagnosis, later time to initiation of palliative intent systemic therapy (4‐8 weeks) for metastatic colorectal and pancreatic cancer does not appear to have a negative impact on survival outcomes.

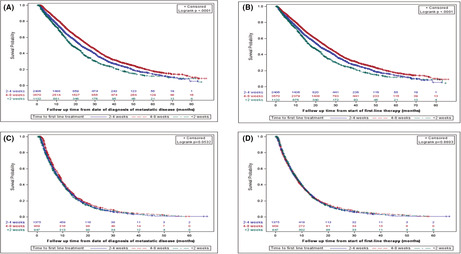

## 1 BACKGROUND

Cancers of the gastrointestinal (GI) system are responsible for a substantial proportion of the cancer burden, globally and in the United States (US).^1^ Together, colorectal cancer (CRC) and pancreatic cancer (PC) represent 11% of all new cancer diagnosis in the United States, and about 17% of all cancer‐related deaths in men and women in the US.^2^ About 60%–70% of patients with PC present with incurable (locally advanced and metastatic disease); and majority of patients with initially resectable tumors will develop recurrence and become incurable.^3^, ^4^, ^5^ On the other hand, colorectal cancer is curable in 70%–80% who present at an earlier stage.^6^, ^7^, ^8^ Nevertheless, up to 40% of these patients may develop recurrent disease and overall survival is estimated at only about 30 months in patients with unresectable disease.^9^, ^10^, ^11^, ^12^

Based on our understanding of tumor kinetics and the natural history of cancer, prompt initiation of therapy is encouraged to obtain optimal survival.^13^, ^14^, ^15^, ^16^ Delay in the initiation of treatment is distressing to patients and physicians, and worldwide, health networks and organizations are dedicated to reducing these delays. In the US, the Institute of Medicine designated timeliness in the delivery of care as a core tenet for assessing effective care delivery.^17^ The impact of delay in cancer diagnosis, surgical intervention, and initiation of adjuvant therapy on overall survival has been extensively documented in the literature for both CRC and PC.^18^, ^19^, ^20^, ^21^, ^22^, ^23^, ^24^, ^25^, ^26^ In summary, early initiation of adjuvant therapy appears to be associated with improvement in OS in CRC while the impact remains unclear in PC, although the weight of evidence suggests that the timing of adjuvant therapy may not be as important as actually administering/receiving adjuvant therapy in PC. Regardless, in the metastatic setting, where the goal of treatment is palliative, there is a scarcity of data to guide physician‐patient interactions around decision making about the optimal time to treatment initiation (TTI), especially its impact on survival in mCRC and mPC. This problem recently became more manifest at the height of the COVID‐19 pandemic which disrupted health care delivery, and forced institutions to provide cancer care on a tier‐system basis^27^, ^28^ with little foundational data to guide recommendations in the palliative setting.

We therefore accessed a real‐world database for a contemporary population of patients with advanced CRC and PC to determine TTI in a population‐based cohort, and to assess the impact of TTI on survival in the real world.

## 2 MATERIALS AND METHODS

### 2.1 Data source

This study used the nationwide de‐identified Flatiron Health longitudinal electronic health record (EHR)‐derived database. During the study period, the de‐identified data originated from ~ 280 US cancer clinics (~ 800 sites of care). The Flatiron Health database is a longitudinal database which includes de‐identified patient‐level structured and unstructured data, curated via technology‐enabled abstraction.^29^, ^30^ Community oncology practice settings make up majority of patients in the database. In the CRC and PC cohort, data about date of initial diagnosis of disease (for recurrent/progressive disease), and date of diagnosis of metastatic disease are collected separately. Therefore, these two variables were available for patients in our cohort who had recurrent CRC or PC. Institutional Review Board approval of the study protocol was obtained prior to study conduct, and included a waiver of informed consent.

### 2.2 Patient population

Based on a diagnosis date between January 2013 and April 2020, we selected patients with mCRC and mPC (de‐novo metastatic disease and recurrent disease) who received at least one line of systemic therapy within 8 weeks of diagnosis of metastatic disease. We selected CRC and PC because they occupy opposite ends of the spectrum of aggressiveness and survival outcomes in GI malignancies in the US. These are also commonly treated cancers, and overall, since around 2010, the first line therapeutic options for both cancers in the US have been stable. We excluded patients who had 1st line therapy documented before date of diagnosis of metastatic disease, those who received 1st line therapy after 8 weeks of diagnosis of metastatic disease, and patients with missing variables which may impact TTI and survival.

### 2.3 Exposure

TTI was defined as time from the date of diagnosis to receipt of any 1st line systemic therapy for mCRC or mPC as documented by the facility. Approximately 70% of patients received what would be considered standard 1st line treatment regimen (single agent and combination agents) based on NCCN recommendations for the period 2014–2020. We hypothesized that most patients in the real‐world would be treated within 4 weeks of diagnosis, and divided TTI into 3 categories (less than 2 weeks, 2–< 4 weeks, and ≥ 4 weeks). Following initial review of the data, the > 4‐week category was divided into 4–8 weeks and > 8 weeks. Thirty‐four percent and 17% of the mCRC and mPC cohorts respectively received 1st treatment > 8 weeks. This subgroup was removed from the final analysis because of our doubts about the veracity of receiving 1st line therapy for metastatic disease more than 8 weeks after diagnosis in the US. We included TTI categories (< 2 weeks, 2–< 4 weeks and 4–8 weeks) in our final analysis.

### 2.4 Variables of interest

We collected clinical demographics, including age at diagnosis, race and sex, date of diagnosis, and insurance status; Insured (Medicare, Medicaid and Private Insurance) versus Other (Self Pay, Patient Assistance Programs, Workers Compensation, ‘Other Payer Unknown, Other Government Program) The American Joint Committee on Cancer (AJCC) clinical stage at initial diagnosis (for recurrent disease) was also abstracted. The date of receipt of first line systemic therapy, the type of therapy received, date of progression, date of receipt of second line therapy, date of death was also collected. Patients were censored at the time of death or at date of last follow‐up visit which is defined as latest date of end of treatment line date and last visit date.

### 2.5 Outcome measures

The primary outcomes were to determine the TTI in a real‐world cohort of mCRC and mPC, RW‐ OS of patients in each TTI category and to evaluate the impact of TTI (based on the 3 defined categories) on RW‐OS. To account for the immortal period between diagnosis and initiation of therapy, we also explored the impact of TTI on post‐chemotherapy overall‐survival (PC‐OS), defined as the date from initiation of first line therapy to date of death. Furthermore, we sought to determine factors that affected TTI. We hypothesized that initiating therapy early (< 2 weeks TTI) would not significantly impact RW‐OS.

### 2.6 Statistical analysis

Demographics and clinical characteristics for the eligible patients were presented by TTI groups. ANOVA or chi‐squared test was used to compare these characteristics among TTI groups. We used adjusted and unadjusted multinomial logistic regression model to evaluate the association of demographic and clinical factors with TTI. The probability of OS and PC‐OS by TTI groups were estimated by using Kaplan–Meier survival analysis. Log rank tests were employed to determine if there is statistically significant association between the survival probabilities based on TTI groups. To estimate the effect of TTI groups on OS, cox proportional hazard models were performed. These were adjusted by demographics and clinical factors. The 2–< 4 weeks TTI category was considered the control group for comparisons. Statistical analyses were performed for mCRC and mPC patients separately by utilizing SAS 9.4(SAS institute). Statistical significance was defined at p < 0.05. Little's test was performed to determine if there is no relationship between the missingness of the data and any values, observed or missing (Missing Completely at Random, MCAR). Multiple imputation analysis was applied for missing data sensitivity analysis. PROC MI and PROC MIANALYZE SAS procedures were performed to generate 10 imputed data by using a separate conditional distribution for each imputed variable and to generate valid statistical inference about estimations by combining results from 10 complete data sets. The primary analysis result was compared with the combined result and confirmed that the presented estimations (complete data analysis) are not sensitive to missing data.

## 3 RESULTS

### 3.1 Population characteristics

**FIGURE 1:**
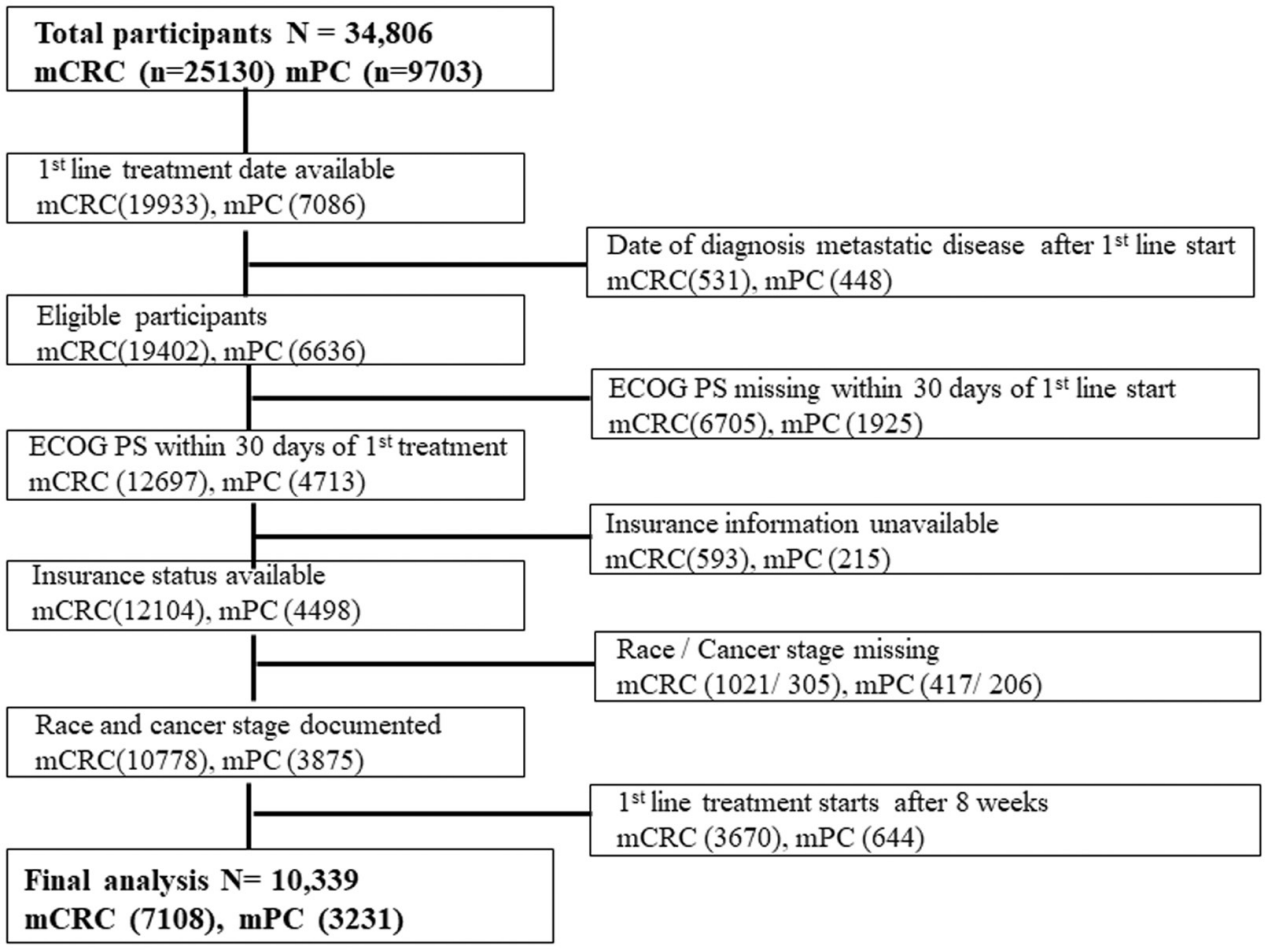
Flow diagram of patients included in the analysis. mCRC: metastatic colorectal cancer, mPC: metastatic pancreatic cancer, ECOG PS: Eastern Cooperative Oncology Group Performance Status.

After applying exclusion criteria (Figure 1), we included 10,339 patients in the final analysis (7108 with mCRC and 3231 with mPC). The median age of patients with mCRC was 64 years (range 20–85) and 68 years for mPC (range 26–85). Fifty‐six percent (56%) of patients in both groups were male, and majority were white, (70% mCRC, 74% mPC). Only 8% in the mCRC group and 5% in the mPC cohort identified as Hispanic/Latino. Table 1 summarizes key demographic and clinical characteristics. The median TTI was 28 days for mCRC and 20 days for mPC (eFigure 1). Majority of mCRC in our analysis were in the 4–8‐week TTI category (50%), whereas most patients with mPC were treated within 2–< 4 weeks (43%) (eTable 1).

**TABLE 1:** Sociodemographic and clinical characteristics

	Metastatic colorectal cancer				Metastatic pancreatic cancer			
Covariates	< 2 weeks *n* = 1132 (%)	2–< 4 weeks *n* = 2406 (%)	4–8 weeks *n* = 3570 (%)	All patients *N* = 7108 (%)	< 2 weeks *n* = 947 (%)	2–< 4 weeks *n* = 1375 (%)	4–8 weeks *n* = 909 (5)	All patients *N* = 3231 (%)
Age^a^								
Median	64	64	64	64	68	69	68	68
Range	23–85	20–85	22–85	20–85	26–85	32–85	28–85	26–85
Sex								14
Female	500 (44.2)	1007 (41.9)	1619 (45.4)	3126 (44)	419 (44.2)	585 (42.5)	425 (46.8)	1429 (44)
Male	632 (55.8)	1399 (58.1)	1951 (54.6)	3982 (56)	528 (55.8)	790 (57.5)	484 (53.2)	1802 (56)
Race								
Blacks	104 (9.2)	241 (10)	387 (10.8)	732 (10.3)	62 (6.5)	130 (9.5)	94 (10.3)	286 (8.9)
Hispanic	78 (6.9)	163 (6.8)	300 (8.4)	541 (7.6)	51 (5.4)	65 (4.7)	58 (6.4)	174 (5.4)
Other	156 (13.8)	291 (12.1)	446 (12.5)	893 (12.6)	115 (12.1)	173 (12.6)	108 (11.9)	396 (12.3)
White	794 (70.1)	1711 (71.1)	2437 (68.3)	4942 (69.5)	719 (75.9)	1007 (73.2)	649 (71.4)	2375 (73.5)
Insurance status^b^								
Insured	1009 (89.1)	2098 (87.2)	3119 (87.4)	6226 (87.6)	856 (90.4)	1216 (88.4)	785 (86.4)	2857 (88.4)
Performance status								
ECOG 0	465 (41.1)	1101 (45.8)	1608 (45)	3174 (44.7)	321 (33.9)	465 (33.8)	286 (31.5)	1072 (33.2)
ECOG 1	492 (43.5)	966 (40.1)	1494 (41.8)	2952 (41.5)	442 (46.7)	641 (46.6)	452 (49.7)	1535 (47.5)
ECOG 2	146 (12.9)	280 (11.6)	377 (10.6)	803 (11.3)	157 (16.6)	217 (15.8)	133 (14.6)	507 (15.7)
ECOG 3	29 (2.6)	59 (2.5)	91 (2.5)	179 (2.5)	27 (2.9)	52 (3.8)	38 (4.2)	117 (3.6)
Stage of at initial diagnosis^c^								
Resectable	623 (55)	985 (40.9)	1083 (30.3)	2691 (37.9)	233 (24.6)	216 (15.7)	151 (16.6)	600 (18.6)
Unresectable	509 (45)	1421 (59.1)	2487 (69.7)	4417 (62.1)	714 (75.4)	1159 (84.3)	758 (83.4)	2631 (81.4)

^a^ Age at diagnosis of metastatic disease.

^b^ Any documentation of insurance status, Insured (Medicare, Medicaid, and Private Insurance), Other (Self Pay, Patient Assistance Programs, Workers Compensation, Other Payer Unknown, Other Government Program).

^c^ Stage of disease at initial diagnosis. Resectable disease (Stages I–III for colorectal cancer, Stages I and II for pancreatic cancer) ECOG: Eastern Cooperative Oncology Group.

### 3.2 Factors associated with TTI

On univariate analysis for mCRC (eTable 2), using the 2–< 4‐weeks TTI as the control, patients who identified as Hispanic/Latinos were more likely to be treated at 4–8 weeks compared to whites (OR 1.29, 95% CI 1.06–1.58, *p* = 0.01). In addition, females were more likely than males to fall into the 4–8‐weeks TTI category (OR 1.15, 95% CI 1.04–1.28, *p* = 0.0076). As expected, patients with recurrent CRC (Stage I‐III at initial diagnosis) were more likely to receive 1st line treatment in less than 2 weeks (OR 1.77, 95% CI 1.53–2.04, *p* < 0.0001). These factors remained significant on multinomial logistic regression modeling (Table 2). Interestingly, patients with ECOG performance status 1 (OR 1.24, 95% CI 1.06–1.45, *p* = 0.006) and 2 (OR 1.32, 95% CI 1.04–1.67, *p* = 0.02) were more likely than those with ECOG 0 to receive 1st line treatment in less than 2 weeks (Table 2).

**TABLE 2:** Multinomial logistic regression analysis of factors associated with time to treatment initiation in metastatic colorectal and pancreatic cancer

Effect	Time to treatment initiation	Odds ratio	95% Wald confidence interval	*p*‐value	Time to treatment initiation	Odds ratio	95% confidence interval	*p*‐value
Metastatic colorectal cancer					Metastatic pancreatic cancer			
Age at diagnosis of metastatic disease								
	< 2 weeks versus 2–4 weeks	0.995	0.989–1.001	0.0919	< 2 weeks versus 2–4 weeks	0.992	0.984–1.001	0.0823
	4–8 weeks versus 2–4 weeks	1.006	1.002–1.01	0.0071	4–8 weeks versus 2–4 weeks	1.003	0.995–1.012	0.4505
Race								
Blacks versus White	< 2 weeks versus 2–4 weeks	0.905	0.707–1.159	0.4305	< 2 weeks versus 2–4 weeks	0.685	0.498–0.943	0.0204
	4–8 weeks versus 2–4 weeks	1.143	0.961–1.361	0.1318	4–8 weeks versus 2–4 weeks	1.109	0.834–1.474	0.4777
Hispanic/Latino versus White	< 2 weeks versus 2–4 weeks	1.034	0.776–1.378	0.8206	< 2 weeks versus 2–4 weeks	1.115	0.761–1.634	0.5769
	4–8 weeks versus 2–4 weeks	1.372	1.119–1.683	0.0024	4–8 weeks versus 2–4 weeks	1.375	0.95–1.99	0.0914
Other versus White	< 2 weeks versus 2–4 weeks	1.138	0.919–1.41	0.2356	< 2 weeks versus 2–4 weeks	0.939	0.727–1.213	0.6308
	4–8 weeks versus 2–4 weeks	1.098	0.934–1.29	0.2582	4–8 weeks versus 2–4 weeks	0.961	0.741–1.247	0.7646
Sex								
Female versus Male	< 2 weeks versus 2–4 weeks	1.110	0.962–1.282	0.1539	< 2 weeks versus 2–4 weeks	1.057	0.893–1.251	0.5187
	4–8 weeks versus 2–4 weeks	1.142	1.028–1.269	0.0135	4–8 weeks versus 2–4 weeks	1.179	0.996–1.397	0.0561
Insurance status^a^								
Insured versus other	< 2 weeks versus 2–4 weeks	0.84	0.669–1.053	0.1304	< 2 weeks versus 2–4 weeks	0.816	0.62–1.074	0.1473
	4–8 weeks versus 2–4 weeks	0.948	0.809–1.111	0.5124	4–8 weeks versus 2–4 weeks	1.195	0.927–1.54	0.1682
Stage at initial diagnosis^b^								
Resectable versus unresectable	< 2 weeks versus 2–4 weeks	1.817	1.571–2.101	< 0.0001	< 2 weeks versus 2–4 weeks	1.724	1.4–2.124	< 0.0001
	4–8 weeks versus 2–4 weeks	0.610	0.547–0.681	< 0.0001	4–8 weeks versus 2–4 weeks	1.061	0.844–1.333	0.611
ECOG performance status								
1 versus 0	< 2 weeks versus 2–4 weeks	1.241	1.063–1.45	0.0064	< 2 weeks versus 2–4 weeks	1.016	0.841–1.227	0.8712
	4–8 weeks versus 2–4 weeks	1.026	0.917–1.149	0.653	4–8 weeks versus 2–4 weeks	1.14	0.941–1.38	0.1802
2 versus 0	< 2 weeks versus 2–4 weeks	1.319	1.044–1.665	0.0201	< 2 weeks versus 2–4 weeks	1.096	0.851–1.412	0.4776
	4–8 weeks versus 2–4 weeks	0.853	0.715–1.018	0.0779	4–8 weeks versus 2–4 weeks	0.975	0.749–1.269	0.8515
3 or more versus 0	< 2 weeks versus 2–4 weeks	1.253	0.789–1.991	0.3393	< 2 weeks versus 2–4 weeks	0.787	0.482–1.284	0.337
	4–8 weeks versus 2–4 weeks	0.964	0.686–1.355	0.8326	4–8 weeks versus 2–4 weeks	1.172	0.751–1.828	0.4857

^a^ Any documentation of insurance status, Insured (Medicare, Medicaid, and Private Insurance), Other (Self Pay, Patient Assistance Programs, Workers Compensation, Other Payer Unknown, Other Government Program).

^b^ Stage of disease at initial diagnosis. Resectable disease (Stages I‐III for colorectal cancer, Stages I and II for pancreatic cancer) ECOG: Eastern Cooperative Oncology Group.

For mPC, univariate analysis (eTable 3) suggested that the odds of Blacks receiving 1st line treatment < 2 weeks was less than Whites receiving treatment within the same TTI category (OR 0.67, 95% CI 0.5–0.92, *p* = 0.013). Females were also more likely than males to be treated within 4–8‐week TTI (OR 1.19 95% CI 1.002–1.4, *p* = 0.048), and those with recurrent disease (Stage I and II at initial diagnosis) were more likely to receive treatment in < 2 weeks (OR 1.75 95% CI 1.42–2.16, *p* < 0.0001).

With multinomial logistic regression, only Black race and stage at initial diagnosis remained associated with TTI (Table 2).

### 3.3 Survival outcomes

**FIGURE 2:**
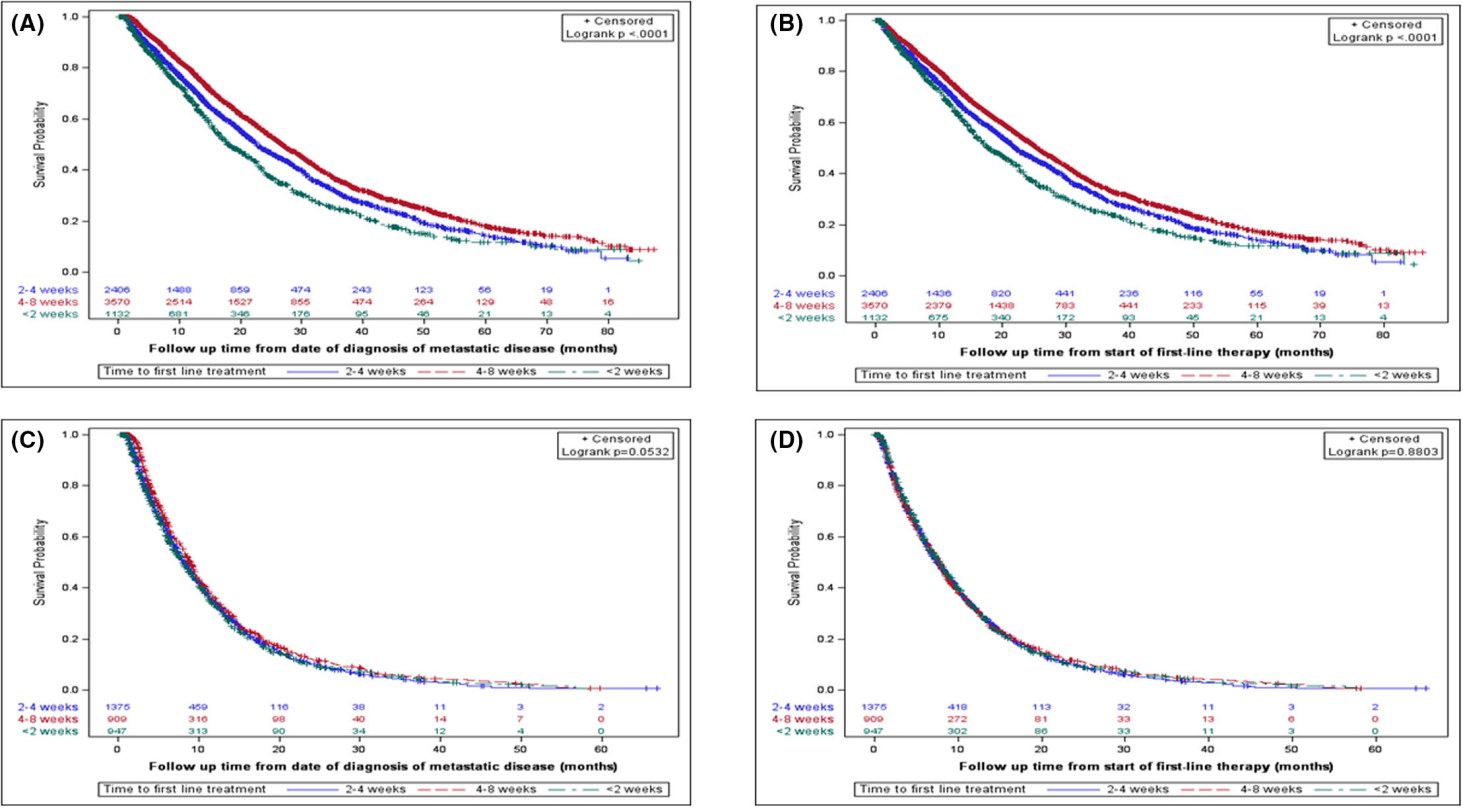
Kaplan–Meier overall survival curves for patients with metastatic colorectal cancer and pancreatic cancer according to time to treatment initiation. (A) Overall survival for metastatic colorectal cancer from date of diagnosis of metastatic disease. (B) Overall survival for metastatic colorectal cancer from date of initiation of treatment (post chemotherapy overall survival). (C) Overall survival for metastatic pancreatic cancer from date of diagnosis of metastatic disease. (D) Overall survival for metastatic pancreatic cancer from date of initiation of treatment (post chemotherapy overall survival)

The median RW‐OS for all patients with mCRC (*n* = 7108) was 24 months. The median RW‐OS in this analysis was highest in the 4–8 week TTI (26.9 months), compared to 22.6 months in the 2–< 4‐week TTI group and 18.05 months for those treated in < 2 weeks (*p* < 0.0001) (Figure 2A). Post‐chemotherapy OS was lower across the categories but remained highest in the 4–8‐week TTI group (25.5 months vs. 22 months in the 2–< 4‐week TTI group, *p* < 0.0001) and lowest in the < 2‐weeks TTI group (Figure 2B).

For mPC, median OS for the entire cohort (*n* = 3231) was 8 months. There was no statistically significant difference among the different TTI groups (*p* = 0.05), although median OS was nominally higher (9 months) in the 4–8‐week category compared to 8 months in the < 2 weeks and 2–< 4‐week TTI groups (Figure 2C). There was also no difference in PC‐OS (*p* = 0.88) among the different groups (7.8 months vs. 7.5 months vs. 7.8 months) (Figure 2D).

### 3.4 Impact of TTI on survival

Cox regression model for adjusted hazards for death was performed to determine the impact of TTI categories on OS (Table 3). After adjusting for other clinical and demographic factors in the model, the 4–8 weeks TTI was associated with a lower hazard of death (HR 0.782, 95% CI 0.73–0.84, *p* < 0.0001) compared to the 2–< 4 weeks group. Conversely, the < 2‐week group was associated with a higher hazard of death (HR 1.26, 95% CI 1.14–1.38, *p* < 0.0001) for patients with mCRC. The pattern was similar for PC‐OS (eTable 4).

**TABLE 3:** Cox Proportional Hazards Model for Overall Survival from time of diagnosis of metastatic disease

	Colorectal cancer		Pancreatic cancer	
	Hazard ratio (95% CI)	*p*‐value	Hazard ratio (95% CI)	*p*‐value
Time to treatment initiation				
< 2 weeks versus 2–4 weeks	1.256 (1.145, 1.379)	< 0.0001	1.048 (0.954, 1.152)	0.327
4–8 weeks versus 2–4 weeks	0.782 (0.728, 0.839)	< 0.0001	0.881 (0.801, 0.969)	0.0094
Age^a^	1.017 (1.014, 1.02)	< 0.0001	1.008 (1.004, 1.012)	0.0003
Race				
Blacks versus White	1.149 (1.037, 1.272)	0.0079	1.022 (0.889, 1.174)	0.7588
Hispanic/Latino versus White	0.933 (0.815, 1.068)	0.3154	0.974 (0.807, 1.174)	0.7793
Other versus White	1.072 (0.971, 1.182)	0.1671	1.025 (0.904, 1.162)	0.7035
Sex				
Female versus Male	0.973 (0.914, 1.037)	0.4046	0.890 (0.822, 0.963)	0.0038
Insurance status^b^				
Insured versus Other	1.173 (1.060, 1.299)	0.0021	1.188 (1.05, 1.344)	0.0064
ECOG performance status				
ECOG 1 versus 0	1.428 (1.332, 1.532)	< 0.0001	1.297 (1.185, 1.420)	< 0.0001
ECOG 2 versus 0	2.447 (2.215, 2.703)	< 0.0001	1.998 (1.772, 2.252)	< 0.0001
ECOG 3 or more versus 0	4.51 (3.776, 5.387)	< 0.0001	2.508 (2.018, 3.116)	< 0.0001
Stage at initial diagnosis^c^				
Resectable versus unresectable	0.780 (0.729, 0.835)	< 0.0001	0.847 (0.765, 0.937)	0.0013

^a^ Age at diagnosis of metastatic disease.

^b^ Any documentation of insurance status, Insured (Medicare, Medicaid, and Private Insurance), Other (Self Pay, Patient Assistance Programs, Workers Compensation, Other Payer Unknown, Other Government Program).

^c^ Stage of disease at initial diagnosis. Resectable disease (Stages I–III for colorectal cancer, Stages I and II for pancreatic cancer) ECOG: Eastern Cooperative Oncology Group.

In mPC, despite similar RW‐OS (and PC‐OS) numbers for the three treatment categories, the cox regression model suggested that the 4–8 weeks TTI category was associated with a lower hazard of death (HR 0.88, 95% CI 0.8–0.97, *p* = 0094). However, compared to the 2–< 4 weeks TTI, the < 2 weeks TTI was not associated with an increased or decreased hazard of death (HR 1.05, 95% CI 0.95–1.15, *p* = 0.33).

## 4 DISCUSSION

There is no rigorously defined threshold for the optimal TTI for mCRC and mPC. While this may elicit the obvious ‘the earlier, the better’ response, the question is not trivial, to the patient or the oncology team. Furthermore, there is limited data on factors that affect TTI and the impact of TTI on survival in patients with metastatic GI malignancies. We report a median TTI of 28 days in a real‐world cohort of patients with mCRC and 20 days for patients with mPC who received treatment within 8 weeks of diagnosis of metastatic disease. Although there were some disparities in TTI for mCRC and mPC, our data suggests that initiating therapy within 2 weeks of diagnosis, was not associated with improved survival outcomes in the population analyzed, and later treatment in 4–8 weeks, was not detrimental to outcomes.

To our knowledge, this is the largest study to assess the TTI in the metastatic setting for CRC and PC in the United States. Lee and colleagues reviewed a large database of Taiwanese patients with Stage I through Stage IV CRC. They recommended an optimal TTI of 30 days for all stages of CRC. This was based on worse survival among patients treated at 31–150 days from diagnosis. In their study, 90% of patients were treated within 30 days. This is in contrast with our data where 50% received 1st line therapy 4–8 weeks after diagnosis. Although they did not provide further granularity within the 30‐day period, they provide a useful benchmark for TTI.^31^ Another hospital‐based dataset from Taiwanese investigators reported a median TTI of 14 days (range 0–163) for mPC. Almost 50% of the 838 patients in this cohort received first line therapy within 2 weeks of diagnosis.^32^ In our US cohort, the corresponding proportion was 30%, with about 70% receiving first line therapy within 4 weeks of diagnosis. This is similar to a French cohort where 50% of patients received therapy for PC (all stages included) within 29 days of diagnosis.^33^ Given the aggressive nature of the disease and the poor overall survival, it is not surprising that mPC is treated more urgently than mCRC.

In mCRC, patients who identified as Hispanics tended to be treated within the latest TTI category. The real significance of this is unclear because only about 8% (*n* = 541) of patients were identified as Hispanic/Latino. Because 50% of patients with mCRC were treated in 4–8 weeks, it would not be unusual for the small Hispanic population size to fall into the largest TTI category. Nevertheless, other investigators have previously reported that Hispanic status increased the likelihood of delay in treatment initiation in CRC compared to Whites. Of note, only 14% of patients in that analysis had Stage IV CRC, with only 1.4% of the CRC population identified as Hispanic.^34^ Racial disparities in CRC treatment and outcomes have been extensively documented with Blacks showing persistently worse outcomes compared to Whites.^35^, ^36^ Differences in TTI in the curative setting have also been described,^37^, ^38^ although when socioeconomic barriers to accessing medical care, including insurance coverage, are removed, as occurs in patients treated within the Military Health System (MHS), the racial differences in TTI are no longer apparent.^39^ In our dataset, there was no significant difference in TTI for mCRC between Blacks and Whites. This was not the case however, in the mPC cohort, where Blacks were less likely to be treated within the < 2 weeks category compared to Whites. We also found that TTI was not significantly affected by health insurance status. This was surprising as we expected a delay in TTI for those without traditional insurance. In this data set, almost 90% of patients had health insurance benefits through Medicare, Medicaid, or a private insurer. This number is consistent with the literature,^40^, ^41^ so it is unlikely that our decision to exclude patients who did not have treatment documented within eight weeks of diagnosis significantly reduced the numbers of those who were uninsured, (or had non‐traditional health insurance) and required financial assistance.

A major highlight of this study was the impact of TTI on OS. Compared to the control TTI category, the < 2 weeks TTI group was associated with worse survival outcomes in mCRC, and there was an increased hazard of death within this group in mPC. This was in line with the hypothesis for this study, previously described as a waiting time paradox.^42^ Oncologists may expedite the treatment of ailing patients, and tailor the standard management plan to accommodate their relative frailty. Conversely, more robust patients are more likely to receive the full suite of necessary diagnostic, palliative, and treatment procedures thus ‘delaying’ treatment. The fact that mCRC patients judged to have an ECOG PS 1 or 2 were more likely to be treated within the < 2‐week period may indirectly lend some credence to this observation. Importantly, despite ECOG PS 1 and 2 being associated with higher odds of receiving treatment within 2 weeks in mCRC, as expected, higher ECOG PS was associated with a higher hazard of death (Table 3). The 4–8‐week TTI category in mCRC was paradoxically associated with better survival outcomes compared to control, with lower hazard of death. Again, the argument above may explain some of this paradox. In addition to patient‐ and treatment‐related factors that contribute to disease outcome, the impact of the biology of the cancer on overall outcomes may be under‐appreciated, as suggested by Esserman and colleagues.^43^ In the metastatic setting, where cancer is always present, and treatment ongoing, it is likely that disease biology, independent of its modification by treatments, plays a role to varying degrees, in determining survival. It is notable that in the more aggressive mPC, outcomes were uniformly dismal, without a notable difference in the survival numbers across the TTI categories. Similarly, in the adjuvant setting, TTI does not appear to impact survival in PC.^24^, ^44^ Overall, it is quite plausible that what we have captured as TTI in this analysis, may itself be a surrogate for oncologists' view of the clinical status of the patient, and aggressiveness (perceived and real) of the disease at the time of diagnosis.

## 5 LIMITATIONS

Although this was a large real‐world data set, we excluded about 60% of the patients in both cohorts due to missing information. We performed tests to confirm that these results are not sensitive to missing data. More importantly, patients who received treatment after 8 weeks (17%–34% of those with otherwise complete data for analysis) were excluded from the final analysis. The Flatiron Health database has a 90‐day gap rule to exclude patients without a documentation of activity (treatment or other) in their EHR during the first 90 days after diagnosis. This is standard practice for publications that have used this database.^45^, ^46^ We also thought it would be unusual to not start palliative intent therapy within 60 days in the US, and we set the 8‐week benchmark as a reasonable compromise for starting palliative intent therapy in the real‐world in the US. The Flatiron Health database 90‐day gap rule likely takes this into consideration. Patients captured as receiving 1st line therapy after 90 days may have started therapy at a facility outside the Flatiron database, then continued treatment at a facility within the database, 90 days after diagnosis. To buttress this point, for those excluded because they received treatment > 8 weeks after diagnosis, in the mCRC cohort, the median TTI was 106 days, and 113 days for mPC. The median OS were 32.3 months and 14.8 months, respectively (data not shown). Given that median survival for untreated mPC is less than three months,^47^, ^48^ the reasoning behind the 90‐day gap rule is sound and our exclusion of these patients from final analysis is also justified. We recognize that this severely limits the generalizability of our data, but we do not think it is proper to include data with questionable veracity in our analysis. On the other hand, about 70% of patients in the database, who had complete data related to this analysis (and fit inclusion and exclusion parameters) were included in this analysis,

In addition, we did not have information about the number and variety of organs involved with metastasis, and whether patients first received chemotherapy in the inpatient setting or otherwise as these data are not available in the database. These may be more objective correlates for clinical fitness, and aggressiveness of disease, at time of diagnosis than age or ECOG performance status and would have provided more robust argument for the wait time paradox hypothesis. We note that investigators have reported the impact of referral to academic centers on treatment initiation delay, and the impact of treatment at such centers on treatment outcomes.^16^, ^43^ We did not think it was necessary to investigate this disparity, as such, we did not include data on treatment facility type. Of note, most patients in the Flatiron Health database originate from community oncology settings; relative community/academic proportions may vary depending on study cohort.

## 6 CONCLUSIONS

This real‐world data analysis, limited to those who initiated treatment within 60 days of diagnosis suggests that in mCRC and mPC, later TTI (up to 8 weeks) may not have a detrimental effect on real‐world survival outcomes. Our analysis, with the limitations of a retrospective study, provides real‐world data for physicians, patients, and patient advocates that may ameliorate some of the anxiety related to the necessary waiting period between diagnosis and initiation of therapy.

## AUTHOR CONTRIBUTIONS

**Olumide Gbolahan**: conceptualization, data curation, formal analysis, investigation, methodology, project administration, supervision, original draft preparation, review, and editing. **Neda Hashemi‐Sadraei:** data curation, formal analysis, methodology, original draft preparation, review and editing. **Suri Yash**: data curation, investigation, project administration, supervision, original draft preparation, review and editing. **Grant Williams:** conceptualization, investigation, methodology, project administration, review and editing. **Rekha Ramachandran:** data curation, formal analysis, investigation, methodology, original draft preparation, review and editing. **Young‐il Kim:** data curation, formal analysis, investigation, methodology, supervision, review and editing. **Ravikumar Paluri:** conceptualization, investigation, original draft preparation, review and editing. **Darryl Outlaw:** data curation, original draft preparation, review and editing. **Basel El‐Rayes:** conceptualization, supervision, original draft preparation, review and editing. **Lisle Nabell:** conceptualization, supervision, original draft preparation, review and editing.

## FUNDING INFORMATION

The authors did not receive any funding for this work.

## ETHICAL APPROVAL STATEMENT

Approval of the study protocol was obtained from the University of Alabama at Birmingham, Institutional Review Board, prior to study conduct, and included a waiver of informed consent.

## Supporting information


**Appendix S1** Supporting Information
eTable 1‐ Proportion of patients with metastatic colorectal and pancreatic cancer treated within the time to treatment initiation (TTI) categories.
eTable 2‐ Univariate analysis of factors associated with time to treatment initiation (TTI) in metastatic colorectal cancer
eTable 3 ‐ Univariate analysis of factors associated with time to treatment initiation (TTI) in metastatic pancreatic cancer
eTable 4: Cox Proportional Hazards Model for overall survival from time of chemotherapy initiation for metastatic disease
eFigure 1: Distribution of time to initiation of first line treatment for patients with metastatic colorectal cancer and pancreatic cancer

## Data Availability

The data that support the findings of this study are available from Flatiron Health Inc. Restrictions apply to the availability of these data, which were used under license for this study. Data are available from the corresponding author and with the permission of Flatiron Health. Interested researchers may contact DataAccess@flatiron.com to determine licensing terms.
