# Supplementary material for: Time to treatment initiation and its impact on real‐world survival in metastatic colorectal cancer and pancreatic cancer
Source: Cancer Med. 2022 Aug 17;12(3):3488–98. doi: 10.1002/cam4.5133 (PMC9939095; doi:10.1002/cam4.5133)
Supplement: Supplementary file 1 — Appendix S1 Supporting Information eTable 1‐ Proportion of patients with metastatic colorectal and pancreatic cancer treated within the time to treatment initiation (TTI) categories. eTable 2‐ Univariate analysis of factors associated with time to treatment initiation (TTI) in metastatic colorectal cancer eTable 3 ‐ Univariate analysis of factors associated with time to treatment initiation (TTI) in metastatic pancreatic cancer eTable 4: Cox Proportional Hazards Model for overall survival from time of chemotherapy initiation for metastatic disease eFigure 1: Distribution of time to initiation of first line treatment for patients with metastatic colorectal cancer and pancreatic cancer [file CAM4-12-3488-s001.docx]

**Supplemental Data**

**eTable 1- Proportion of patients with metastatic colorectal and pancreatic cancer treated within the time to treatment initiation (TTI) categories.**

| **Disease group**  **N (10,339)** | **< 2 weeks**  **n (%)** | **2-<4 week**  **n (%)** | **4-8 weeks**  **n (%)** |
| --- | --- | --- | --- |
| **Colorectal cancer (7,108)** | 1132 (16) | 2406 (34) | 3570 (50) |
| **Pancreatic cancer (3,231)** | 947 (29) | 1375 (43) | 909 (29) |

**eTable 2- Univariate analysis of factors associated with time to treatment initiation (TTI) in metastatic colorectal cancer**

|  | Time to Treatment Initiation | Odds Ratio | 95% confidence interval | | p-value |
| --- | --- | --- | --- | --- | --- |
| Age at diagnosis of metastatic disease | | | | | |
|  | <2 weeks vs 2-4 weeks | 1.001 | 0.995 | 1.006 | 0.8423 |
|  | 4-8 weeks vs 2-4 weeks | 1.002 | 0.998 | 1.006 | 0.3202 |
| Race | | | | | |
| Blacks vs White | <2 weeks vs 2-4 weeks | 0.930 | 0.728 | 1.188 | 0.5609 |
|  | 4-8 weeks vs 2-4 weeks | 1.127 | 0.949 | 1.339 | 0.1725 |
| Hispanic/Latino vs White | <2 weeks vs 2-4 weeks | 1.031 | 0.777 | 1.368 | 0.8314 |
|  | 4-8 weeks vs 2-4 weeks | 1.292 | 1.057 | 1.579 | 0.0122 |
| Other vs White | <2 weeks vs 2-4 weeks | 1.155 | 0.935 | 1.428 | 0.182 |
|  | 4-8 weeks vs 2-4 weeks | 1.076 | 0.917 | 1.263 | 0.3695 |
| Sex | | | | | |
| Female vs Male | <2 weeks vs 2-4 weeks | 1.099 | 0.953 | 1.268 | 0.1938 |
|  | 4-8 weeks vs 2-4 weeks | 1.153 | 1.039 | 1.28 | 0.0076 |
| ^a^Insurance status | | | | | |
| Insured vs Other | <2 weeks vs 2-4 weeks | 0.830 | 0.665 | 1.037 | 0.101 |
|  | 4-8 weeks vs 2-4 weeks | 0.985 | 0.843 | 1.15 | 0.8481 |
| ^b^Stage at initial diagnosis | | | | | |
| Resectable vs Unresectable | <2 weeks vs 2-4 weeks | 1.766 | 1.531 | 2.036 | <.0001 |
|  | 4-8 weeks vs 2-4 weeks | 0.628 | 0.564 | 0.700 | <.0001 |
| ECOG Performance Status | | | | | |
| 1 vs 0 | <2 weeks vs 2-4 weeks | 1.206 | 1.034 | 1.406 | 0.0167 |
|  | 4-8 weeks vs 2-4 weeks | 1.059 | 0.947 | 1.184 | 0.3139 |
| 2 vs 0 | <2 weeks vs 2-4 weeks | 1.235 | 0.983 | 1.55 | 0.0695 |
|  | 4-8 weeks vs 2-4 weeks | 0.922 | 0.776 | 1.096 | 0.3558 |
| 3 or more vs 0 | <2 weeks vs 2-4 weeks | 1.164 | 0.737 | 1.839 | 0.5158 |
|  | 4-8 weeks vs 2-4 weeks | 1.056 | 0.754 | 1.478 | 0.7507 |

a: Any documentation of insurance status, Insured (Medicare, Medicaid, and Private Insurance) Uninsured (Self Pay, Patient Assistance Programs, Workers Compensation, Other Payer Unknown, Other Government Program) b: Stage of disease at initial diagnosis. Resectable disease (Stages I-III for colorectal cancer, Stages I and II for pancreatic cancer) ECOG: Eastern Cooperative Oncology Group

**eTable 3 - Univariate analysis of factors associated with time to treatment initiation (TTI) in metastatic pancreatic cancer**

|  | **Time to treatment initiation** | **Unadjusted Odds ratio** | **95% Wald Confidence Limits** | | **P-value** |
| --- | --- | --- | --- | --- | --- |
| Age at diagnosis of metastatic disease | | | | | |
|  | <2 weeks vs 2-4 weeks | 0.994 | 0.985 | 1.002 | 0.1435 |
|  | 4-8 weeks vs 2-4 weeks | 1.003 | 0.994 | 1.012 | 0.5171 |
| Race | | | | | |
| Blacks vs White | <2 weeks vs 2-4 weeks | 0.668 | 0.487 | 0.918 | 0.0127 |
|  | 4-8 weeks vs 2-4 weeks | 1.122 | 0.845 | 1.489 | 0.426 |
| Hispanic/Latino vs White | <2 weeks vs 2-4 weeks | 1.099 | 0.752 | 1.605 | 0.6256 |
|  | 4-8 weeks vs 2-4 weeks | 1.385 | 0.959 | 1.999 | 0.0827 |
| Other vs White | <2 weeks vs 2-4 weeks | 0.931 | 0.722 | 1.201 | 0.5819 |
|  | 4-8 weeks vs 2-4 weeks | 0.969 | 0.747 | 1.256 | 0.8101 |
| Sex | | | | | |
| Female vs Male | <2 weeks vs 2-4 weeks | 1.072 | 0.907 | 1.266 | 0.4166 |
|  | 4-8 weeks vs 2-4 weeks | 1.186 | 1.002 | 1.403 | 0.0475 |
| ^a^Insurance status | | | | | |
| Insured vs Other | <2 weeks vs 2-4 weeks | 0.813 | 0.619 | 1.067 | 0.1359 |
|  | 4-8 weeks vs 2-4 weeks | 1.208 | 0.940 | 1.553 | 0.1406 |
| ECOG Performance status | | | | | |
| 1 vs 0 | <2 weeks vs 2-4 weeks | 0.999 | 0.829 | 1.204 | 0.9906 |
|  | 4-8 weeks vs 2-4 weeks | 1.146 | 0.948 | 1.387 | 0.159 |
| 2 vs 0 | <2 weeks vs 2-4 weeks | 1.048 | 0.816 | 1.345 | 0.7126 |
|  | 4-8 weeks vs 2-4 weeks | 0.997 | 0.767 | 1.294 | 0.979 |
| 3 or more vs 0 | <2 weeks vs 2-4 weeks | 0.752 | 0.463 | 1.223 | 0.251 |
|  | 4-8 weeks vs 2-4 weeks | 1.188 | 0.763 | 1.851 | 0.4462 |
| ^b^Stage at initial diagnosis | | | | | |
| Resectable vs unresectable | <2 weeks vs 2-4 weeks | 1.751 | 1.423 | 2.155 | <.0001 |
|  | 4-8 weeks vs 2-4 weeks | 1.069 | 0.852 | 1.342 | 0.5648 |

a: Any documentation of insurance status, Insured (Medicare, Medicaid, and Private Insurance), Other (Self Pay, Patient Assistance Programs, Workers Compensation, Other Payer Unknown, Other Government Program) b: Stage of disease at initial diagnosis. Resectable disease (Stages I-III for colorectal cancer, Stages I and II for pancreatic cancer) ECOG: Eastern Cooperative Oncology Group

**eTable 4: Cox Proportional Hazards Model for overall survival from time of chemotherapy initiation for metastatic disease**

|  | Colorectal cancer | | Pancreatic cancer | |
| --- | --- | --- | --- | --- |
|  | Hazard Ratio (95% CI) | p-value | Hazard Ratio (95% CI) | p-value |
| Time to treatment initiation |  |  |  |  |
| <2 weeks vs 2-4 weeks | 1.215 (1.107 - 1.334) | <.0001 | 1.001 (0.911 - 1.1) | 0.984 |
| 4-8 weeks vs 2-4 weeks | 0.807 (0.753 - 0.866) | <.0001 | 0.881 (0.866 – 1.048) | 0.3185 |
| Age^1^ | 1.016 (1.013 - 1.019) | <.0001 | 1.008 (1.003 - 1.012) | 0.0003 |
| Race |  |  |  |  |
| Blacks vs White | 1.143 (1.032 - 1.266) | 0.0105 | 1.019 (0.887 - 1.171) | 0.7859 |
| Hispanic/Latino vs White | 0.92 (0.804 - 1.053) | 0.2244 | 0.974 (0.807, 1.174) | 0.7793 |
| Other vs White | 1.07(0.97 - 1.180) | 0.1785 | 1.02 (0.9 - 1.157) | 0.7524 |
| Sex |  |  |  |  |
| Female vs Male | 0.971 (0.911 - 1.034) | 0.3558 | 0.89 (0.822 - 0.963) | 0.0038 |
| Insurance status^2^ |  |  |  |  |
| Insured vs Other | 1.115 (1.043 - 1.279) | 0.0055 | 1.2 (1.06 - 1.357) | 0.0039 |
| ECOG Performance status |  |  |  |  |
| ECOG 1 vs 0 | 1.418 (1.332 - 1.520) | <.0001 | 1.3 (1.188 - 1.423) | <.0001 |
| ECOG 2 vs 0 | 2.417 (2.188 - 2.671) | <.0001 | 1.994 (1.768 - 2.248) | <.0001 |
| ECOG 3 or more vs 0 | 4.34 (3.632 - 5.168) | <.0001 | 2.507 (2.018 - 3.115) | <.0001 |
| Stage at initial diagnosis^3^ | | | | |
| Resectable vs unresectable | 1.344 (1.244 -1.452) | <.0001 | 0.848 (0.766 - 0.938) | 0.0014 |

1: age at diagnosis of metastatic disease 2 Any documentation of insurance status, Insured (Medicare, Medicaid, and Private Insurance), Other (Self Pay, Patient Assistance Programs, Workers Compensation, Other Payer Unknown, Other Government Program) 3: Stage of disease at initial diagnosis. Resectable disease (Stages I-III for colorectal cancer, Stages I and II for pancreatic cancer) ECOG: Eastern Cooperative Oncology Group

**eFigure 1: distribution of time to initiation of first line treatment for patients with metastatic colorectal cancer and pancreatic cancer**
